# Health and lifestyle factors associated with sexual difficulties in men – results from a study of Australian men aged 18 to 55 years

**DOI:** 10.1186/s12889-016-3705-6

**Published:** 2016-10-31

**Authors:** Marisa Schlichthorst, Lena A. Sanci, Jane S. Hocking

**Affiliations:** 1 Centre for Mental Health, Melbourne School of Population and Global Hralth, The University of Melbourne, Melbourne, 3010 Australia; 2 Department of General Practice, The University of Melbourne, Melbourne, 3010 Australia

## Abstract

**Background:**

Sexual difficulties (SD) are common among men of all ages and can have considerable impact on quality of life and indications for future health. SD are associated with mental and physical wellbeing and with relationship satisfaction, yet they are rarely discussed with medical professionals who are often ill equipped to assess and manage them. This paper provides an updated overview on the status of SD in Australian men from 18 to 55 years of age and will form a baseline comparison for future analyses of SD based on Ten to Men data.

**Methods:**

We used data from Ten to Men, the Australian Longitudinal Study on Male Health. SD was measured using eight items capturing specific sexual difficulties. We examined associations of a range of health and lifestyle factors (smoking, alcohol consumption, illicit drug use, obesity and new sexual partners, self-rated health status, disability, pain medication, diagnosed physical and mental health conditions) with each SD using logistic regression. The sample included 12,636 adult males who had previously been sexually active. Analysis was stratified by age (18–34 years versus 35–55 years).

**Results:**

This paper shows that experiencing SD is relatively common among Australian men – overall half the sample (54 %; 95 % CI: 0.53–0.55) experienced at least one SD for more than 3 months over the past 12 months. While more common in older men aged 45 to 55 years, almost half the 18 to 24 year old men (48 %) also reported at least one SD highlighting that SD affects men of all ages. We found that SDs were associated with both lifestyle and health factors, although the strongest associations were observed for health factors in both age groups, in particular poor self-rated health, having a disability and at least one mental health condition. Lifestyle factors associated with SDs in men of all ages included smoking, harmful alcohol consumption and drug use in the past 12 months. Obesity was only associated with an increased rate of SD in men aged 35 to 55 years.

**Conclusion:**

Sexual difficulties are common among men of all ages and increasingly more prevalent as men grow older. They are strongly associated with both health and lifestyle factors. With previous literature showing that SDs can be a precursor of an underlying or developing physical and mental health condition, it is imperative that sexual health and sexual functioning is discussed with a doctor as part of a standard health check and across the lifespan.

## Background

Sexual difficulties are common among men and can have considerable impact on their quality of life [1]. In men, sexual dysfunction is a syndrome that includes one or more of the following sexual difficulties: lack or loss of sexual desire, sexual aversion and lack of sexual enjoyment, erectile dysfunction, orgasmic dysfunction and premature ejaculation (PE) [2]. Sexual dysfunction is a clinical diagnosis made when sexual difficulties or problems are persistent and recurrent over time and when they cause significant personal or interpersonal distress, and not merely transitory fluctuations in sexual function [3]. In this paper, we use the term sexual difficulties to refer to a range of sexual conditions reported by men that can impact on their sexual satisfaction [4].

Several studies have investigated the prevalence and factors associated with sexual difficulties and while the definitions of sexual difficulties used in these studies have varied considerably, the evidence suggests that they are common among men, increase with age and can have considerable impact on men’s quality of life [1, 4–9]. Recent population data from the UK, US and Europe have found that the most commonly experienced sexual difficulties are lack of interest in sex, reaching climax more quickly than desired and difficulty in getting or sustaining an erection. Similar trends are found in Australia with population-based data finding that lack of interest in sex was the most commonly reported SD in men aged 16 to 55 years (25 %) following by reaching climax more quickly than desired (24 %); difficulty in getting or sustaining an erection was reported by 10 % [1].

Sexual difficulties (hereafter referred to as SD) can be related to mental and physical wellbeing and relationship satisfaction [8, 10–12] and several lifestyle factors have been found to be associated with them. Physical health factors include heart disease, hypertension, stroke, diabetes, obesity, self-rated overall physical health, and anxiety and depression [3, 8, 13–22]. Lifestyle factors include smoking, drinking, sedentary lifestyle and physical activity levels [14, 17, 18, 23–27]. Unfortunately, research investigating factors associated with SD is often limited to cross-sectional studies making it challenging to tease out temporal relationships between risk factors and SD. A comprehensive review of prevalence and incidence data for SD among men and women published in 2010 found only five population-based studies that reported longitudinal data [5]; four of these studies focused exclusively on erectile dysfunction [28–32]; two studies extend to cover various sexual difficulties [33, 34]. As many health conditions such as depression are managed with medications that can impact on sexual function [35], longitudinal data are needed to explore temporal relationships to establish whether it is the health condition or the medication that causes the SD [3]. This is important for clinical management of the health conditions.

While Australian population-based SD data have been previously reported [8, 34], they were collected as part of a national sexual behaviour survey and only limited data about other health and lifestyle factors including co-morbidities were collected. We report here an overview on the status of SD in Australian men from the first wave of data collection from a national population based longitudinal study, Ten to Men. Ten to Men captures a range of health outcomes, health behaviours and related risk factors providing the opportunity to investigate the association of several different lifestyle and health factors with SD. These data will form a baseline comparison for future analyses of SD in Ten to Men, allowing us to investigate causal associations over time.

## Methods

### Participants

We used data from Ten to Men (the Australian Longitudinal Study on Male Health) a population based cohort study capturing a range of health outcomes, health behaviours and related risk factors (including social determinants). The study methodology has been fully described by Currier and colleagues in this supplement of BMC Public Health [36]. Briefly, participants were recruited from households between October 2013 and July 2014 using a stratified, multi-stage, random cluster sampling design. Households were stratified by geographical areas based on the Australian Statistical Geographical Standards (ASGS) and recruited within areas classified as major cities (accounting for 70 % of the Australian population), inner regional (18 %) and outer regional (9 %) areas. We oversampled men from inner and outer regional areas to ensure we had sufficient numbers for statistical analysis within different regional strata.

Men and boys over 14 years of age were asked to complete a detailed hard-copy questionnaire that covered a range of health outcomes, behaviours and related risk factors. The final sample consists of 15,988 male participants aged between 10 and 55 years corresponding to a response fraction of 35 % of those males who were confirmed in-scope. A comparison of Ten to Men participants with the Australian population of males aged 10–55 in the 2011 Census, shows that the Ten to Men cohort are older, more likely to be Australian born, and more likely to live in regional areas reflecting the sample design [36].

For the purpose of this paper, only men aged 18 and over who had ever had sex were included in the analysis, leaving a total sample of 12,636 men. Sex in the context of this study was defined as vaginal, oral or anal sex. Men were included in this analysis regardless of whether or not they reported sex with a partner in the last 12 months because our survey did not differentiate between masturbation and partnered sex. The socio-demographic characteristics of the analysis sample were as follows: 58 % were in major cities, 23 % in inner regional and 19 % in outer regional areas; 12 % were aged 18 to 24 years, 23 % were 25 to 34 years, 31 % were 35 to 44 years and 34 % were 45 to 55 years; 77 % were born in Australia; 60 % completed year 12; 70 % were married or in a de-facto relationship; 86 % were in a sexual relationship at time of survey; 94 % identified as heterosexual and; 87 % were employed.

### Measures

We assessed sexual function using components of the validated sexual function questionnaire developed by the National Survey of Sexual Attitudes and Lifestyles and as used in NATSAL3 [37]. Participants were asked if they had experienced any of eight sexual difficulties for a period of 3 months or longer in the 12 months prior to the study: lacking interest in having sex, lacking enjoyment in sex, feeling anxious during sex, feeling physical pain as a result of sex, feeling no excitement or arousal during sex, not being able to reach climax or taking too long, reaching climax too quickly, having difficulties getting or keeping an erection. Response options to these questions were binary yes/no.

A range of physical health, mental health and lifestyle factors were investigated for their association with experiencing sexual difficulties. Lifestyle factors captured participants’ status as current smoker (i.e., smoker versus non-smoker); harmful or hazardous levels of alcohol consumption (derived from the Alcohol Use Disorder Identification Test - AUDIT) [38]; having used drugs in the past 12 months aggregated into four categories: none, marijuana only, marijuana and other drugs, other drugs only; and obesity (derived from the body mass index (BMI) and categorised as obese with BMI greater or equal to 30 and non-obese with BMI less than 30).

Physical health status was measured by five variables. We used the first questions of the SF12 Health Survey [39] to measure self-rated general health. The original 5-point scale was reduced to a 4-point scale by collapsing the bottom two categories (fair and poor health). A disability score was generated using the Washington Group Disability Scale grouping participants into those with disability and those without disability. [40] A binary variable was generated to capture daily use of pain medication as an indicator for chronic pain. To control for the presence of pre-existing health conditions, a binary variable was derived as an aggregate across 15 high prevalence health conditions. This variable indicates if a participant was diagnosed with at least one condition in the past 12 months. We also derived a binary measure to control for the diagnosis of a mental health conditions in the past 12 months as an aggregate over four conditions, i.e. depression, anxiety, Post Traumatic Stress Disorder (PTSD) and Schizophrenia.

Socio-demographic variables included were age (coded as 18 to 24 years, 25 to 34 years, 35 to ﻿44 years and 45 to 55 years OR as 18 to 34 years and 35 to 55 years); country of birth (coded as either born in Australia or overseas); sexuality (coded as either heterosexual and non-heterosexual); new sexual partners in past 12 months (binary) and; relationship status (coded as in a regular sexual relationship or no relationship at time of the survey).

### Statistical analysis

Proportions and 95 % confidence intervals were calculated for each SD separately and the mean and median of SDs reported per participant were calculated. We assessed trends by age groups using the Score test. For the more common SDs reported by more than 10 % of participants (lacked interest in having sex, lacked enjoyment, felt anxious during sex, did not reach climax or took a long time, reached climax too quickly, had trouble getting or keep an erection), we conducted logistic regression to investigate the association of each health and lifestyle factor with the individual SD. Due to interaction effects between age and our health and lifestyle factors of interest, all analyses were stratified by age group (18 to 34 years versus 35 to 55 years). Each health and lifestyle factor was investigated in a separate model adjusting for socio-demographic variables including sexuality, relationship status, new sexual partner in past 12 months and country of birth. All analyses were performed using Stata/IC 13.1.

## Results

**Fig. 1:**
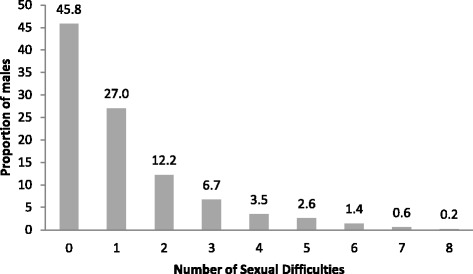
Distribution of total count of SDs per participant

Of the 12,636 men available for analysis, over half (54 %; 95 % CI: 53.6–55.3) experienced at least one sexual difficulty for more than 3 months over the past 12 months (Fig. 1). The most commonly reported SD was ‘reaching climax too early’ (37.2 %; 95 % CI: 36.4–38.1). Feeling no excitement and feeling pain as a result of having sex were the least reported (6 %; 95 % CI: 5.5–6.4 and 3.7 %; 95 % CI: 3.4–4.0 - respectively). The proportion reporting SDs increased with age for all SDs except “did not reach climax or took a long time”. On average men reported having 1.12 SDs (SD = 1.5; 95 % CI: 1.09–1.15) with a median of 1 (Table 1).

**Table 1:** Associations of reported sexual difficulties with age

SD for 3 months or longer over the past 12 months	Total Yes	18–24 years	25–34 years	35–44 Years	45–55 years	Trend^a^
	% Yes (N) 95 % CI	% Yes 95 % CI	% Yes 95 % CI	% Yes 95 % CI	% Yes 95 % CI	*p*-value
Lacked interest in having sex	17.3 (2121) 16.6–17.9	14.6 12.8–16.5	15.1 13.8–16.5	16.7 15.5–17.9	20.2 19.0–21.5	*p* < 0.001
Lacked enjoyment in sex	10.1 (1229) 9.6–10.6	8.1 6.7–9.6	9.5 8.4–10.6	9.2 8.3–10.2	12.1 11.1–13.1	*p* < 0.001
Felt anxious during sex	10.9 (1328) 10.4–11.5	10.3 8.8–12.0	10.2 9.1–11.4	10.7 9.7–11.7	11.8 10.8–12.8	*p* = 0.039
Felt physical pain as a result of sex	3.7 (448) 3.4–4.0	5.0 3.9–6.2	4.1 3.4–4.9	3.2 2.6–3.8	3.4 2.8–4.0	*p* = 0.004
Felt no excitement or arousal during sex	6.0 (724) 5.5–6.4	6.1 4.9–7.4	4.5 3.8–5.3	5.5 4.8–6.3	7.4 6.6–8.2	*P* < 0.001
Did not reach climax or took a long time	15.0 (1817) 14.3–15.6	16.8 14.9–18.8	13.3 12.1–14.6	13.8 12.7–14.9	16.6 15.4–17.7	*p* = 0.137
Reached climax too quickly	37.2 (4527) 36.4–38.1	31.5 29.1–33.9	36.3 34.5–38.1	39.2 37.6–40.8	38.0 36.5–39.5	*p* < 0.001
Had trouble getting or keeping an erection	13.7 (1671) 13.1–14.3	10.8 9.2–12.5	8.1 7.1–9.2	12.3 11.2–13.3	19.9 18.7–21.2	*p* < 0.001
At least one SD over the past 12 months	54.2 (6688) 53.3–55.1	48.3 45.7–50.1	51.6 50.0–53.7	54.2 53.0–56.2	56.6 55.6–58.9	*p* < 0.001
SD-index (# sexual difficulties)						
Mean (SD) 95 % CI	1.12 (1.49) 1.09–1.14	1.02 (1.48) 0.95–1.10	1.0 (1.39) 0.95–1.05	1.08 (1.43) 1.04–1.32	1.26 (1.60) 1.231–1.31	*p* < 0.001

N total sample = 12,636

^a^Score test for binary SD items; non parametric test for trend for SD count variable

### Factors associated with each sexual difficulty

Among lifestyle factors, smoking was associated with increased odds of each SD with the exception of reaching climax too quickly in ﻿35 to 55 year olds and feeling anxious during sex in 18 to 34 years olds. Alcohol consumption was associated with increased odds of each SD in both age groups with the exception of lacked interest in sex among 18 to 34 year olds and feeling anxious during sex across all ages. Drug use was associated with increased odds of each SD across both age groups with the exception of lacked interest in sex among 35 to 55 year olds. Associations differed for the type of drug consumed. Generally, having an SD was more likely for poly drug use (marijuana + other drug use). Obesity was associated with increased SD in older men, but was not associated with any of the SD among 18 to 34 year olds (Tables 2 and 3).

**Table 2:** Correlates of reporting a sexual difficulty for at least 3 months in the last year – psychological SDs

	Lacked interest		Lacked enjoyment		Felt anxious during sex	
	18–34 years	35–55 years	18–34 years	35–55 years	18–34 years	35–55 years
	(*n* = 2925)	(*n* = 5526)	(*n* = 2923)	(*n* = 5500)	(*n* = 2920)	(*n* = 5496)
Lifestyle factors	OR (95 % CI)	OR (95 % CI)	OR (95 % CI)	OR (95 % CI)	OR (95 % CI)	OR (95 % CI)
Currently smoking						
Yes	1.46 (1.15–1.86)	1.60 (1.35–1.89)	1.75 (1.30–2.34)	1.44 (1.16–1.78)	1.18 (0.89–1.56)	1.33 (1.08–1.64)
No	-	-	-	-	-	-
Alcohol consumption						
Harmful consumption	1.18 (0.95–1.45)	1.45 (1.26–1.67)	1.57 (1.20–2.06)	1.28 (1.07–1.53)	1.22 (0.96–1.56)	1.18 (1.00–1.40)
Non-harmful consumption	-	-	-	-	-	-
Drug use in past 12 months	*P* = 0.023	*P* = 0.236	*P* = 0.034	*P* = 0.146	*P* = 0.026	*P* = 0.088
No drugs	-	-	-	-	-	-
Marijuana only	1.30 (0.98–1.72)	1.14 (0.91–1.41)	1.17 (0.81–1.69)	1.00 (0.76–1.34)	1.40 (1.02–1.92)	1.12 (0.86–1.47)
Marijuana and other drugs	1.37 (1.00–1.88)	1.34 (0.96–1.87)	1.71 (1.18–2.49)	1.30 (0.86–1.96)	1.43 (1.00–2.04)	1.56 (1.07–2.28)
Other drugs only	1.69 (1.07–2.65)	1.20 (0.71–2.03)	1.43 (0.78–2.60)	1.78 (1.01–3.13)	1.71 (1.02–2.85)	1.37 (0.75–2.48)
Obesity						
Yes	0.97 (0.74–1.28)	1.50 (1.29–1.74)	1.10 (0.79–1.54)	1.37 (1.14–1.65)	0.94 (0.69–1.29)	1.16 (0.97–1.39)
No	-	-	-	-	-	-
Health Factors	OR (95 % CI)	OR (95 % CI)	OR (95 % CI)	OR (95 % CI)	OR (95 % CI)	OR (95 % CI)
Self-rated health	*P* < 0.001	*P* < 0.001	*P* < 0.001	*P* < 0.001	*P* = 0.008	*P* < 0.001
Excellent	-	-	-	-	-	-
Very good	1.44 (1.08–1.93)	1.60 (1.27–2.01)	1.22 (0.84–1.77)	1.31 (0.98–1.74)	1.45 (1.05–2.01)	1.41 (1.07–1.85)
Good	1.84 (1.35–2.50)	2.36 (1.86–2.98)	1.90 (1.29–2.78)	1.91 (1.43–2.55)	1.56 (1.10–2.22)	2.01 (1.52–2.65)
Fair or poor	3.89 (2.57–5.89)	4.70 (3.55–6.22)	3.39 (2.03–5.64)	4.23 (3.03–5.90)	2.28 (1.38–3.77)	3.34 (2.40–4.65)
Daily pain self-medication						
Yes	1.43 (0.88–2.31)	1.97 (1.61–2.41)	1.08 (0.56–2.10)	1.57 (1.22–2.03)	1.12 (0.62–2.02)	1.29 (1.00–1.67)
No	-	-	-	-	-	-
Disability						
With disability	2.38 (1.65–3.42)	2.28 (1.77–2.92)	3.14 (2.08–4.73)	2.84 (2.15–3.76)	1.90 (1.24–2.91)	2.20 (1.66–2.94)
Without disability	-	-	-	-	-	-
Diagnosed physical health condition in past 12 months						
1 or more conditions diag.	1.35 (1.07–1.72)	1.63 (1.42–1.87)	1.22 (0.90–1.66)	1.58 (1.32–1.88)	1.46 (1.11–1.90)	1.69 (1.43–2.00)
None diagnosed	-	-	-	-	-	-
Diagnosed mental health condition in past 12 months						
1 or more conditions diag.	2.66 (2.07–3.42)	2.72 (2.31–3.21)	3.22 (2.38–4.35)	2.67 (2.19–3.26)	2.77 (2.09–3.66)	2.79 (2.30–3.38)
None diagnosed	-	-	-	-	-	-

Logistic regression models, each model is adjusted for relationship status, sexuality and country of birth; models are stratified by age

**Table 3:** Correlates of reporting a sexual difficulty for at least 3 months in the last year – organic SDs

	Did not reach climax or took a long time		Reached climax too quickly		Trouble getting or keeping an erection	
	18–34 years	35–55 years	18–34 years	35–55 years	18–34 years	35–55 years
	(*n* = 2920)	(*n* = 5496)	(*n* = 2922)	(*n* = 5504)	(*n* = 2919)	(*n* = 5514)
Lifestyle factors	OR (95 % CI)	OR (95 % CI)	OR (95 % CI)	OR (95 % CI)	OR (95 % CI)	OR (95 % CI)
Currently smoking						
Yes	1.49 (1.17–1.88)	1.50 (1.24–1.80)	1.36 (1.13–1.64)	1.08 (0.93–1.25)	1.83 (1.38–2.41)	1.61 (1.35–1.92)
No	-	-	-	-	-	-
Alcohol consumption						
Harmful consumption	1.60 (1.30–1.98)	1.45 (1.24–1.69)	1.59 (1.36–1.86)	1.39 (1.24–1.56)	1.63 (1.26–2.11)	1.25 (1.08–1.46)
Non-harmful consumption	**-**	**-**	-	-	-	-
Drug use in past 12 months	*P* < 0.001	*P* < 0.001	*P* < 0.001	*P* = 0.029	*P* < 0.001	*P* = 0.007
No drugs	-	-	-	-	-	-
Marijuana only	1.38 (1.04–1.83)	1.39 (1.11–1.74)	1.39 (1.12–1.72)	1.03 (0.86–1.24)	2.00 (1.43–2.78)	1.10 (0.87–1.39)
Marijuana and other drugs	1.85 (1.38–2.48)	1.86 (1.33–2.60)	1.83 (1.44–2.32)	1.24 (0.93–1.65)	3.07 (2.20–4.28)	1.61 (1.15–2.25)
Other drugs only	2.10 (1.36–3.23)	2.17 (1.34–3.51)	1.56 (1.08–2.25)	1.78 (1.67–2.71)	2.61 (1.57–4.36)	1.76 (1.07–2.89)
Obesity						
Yes	0.78 (0.59–1.03)	1.34 (1.14–1.57)	1.04 (0.85–1.27)	1.11 (0.98–1.26)	1.12 (0.82–1.54)	1.60 (1.37–1.86)
No	-	-	-	-	-	-
Health Factors	OR (95 % CI)	OR (95 % CI)	OR (95 % CI)	OR (95 % CI)	OR (95 % CI)	OR (95 % CI)
Self-rated health	*P* = 0.002	*P* < 0.001	*P* < 0.001	*P* < 0.001	*P* < 0.001	*P* < 0.001
Excellent	-	-	-	-	-	-
Very good	1.29 (0.98–1.70)	1.30 (1.03–1.65)	1.18 (0.97–1.43)	1.27 (1.09–1.48)	1.01 (0.72–1.42)	1.68 (1.30–2.17)
Good	1.61 (1.20–2.16)	2.04 (1.60–2.59)	1.48 (1.20–1.84)	1.41 (1.20–1.66)	1.67 (1.18–2.36)	2.59 (2.00–3.34)
Fair or poor	1.99 (1.28–3.10)	2.91 (2.16–3.93)	1.99 (1.40–2.84)	1.43 (1.14–1.81)	2.04 (1.22–3.40)	5.05 (3.74–6.82)
Daily pain self-medication						
Yes	1.41 (0.87–2.27)	1.55 (1.24–1.94)	1.30 (0.89–1.91)	1.05 (0.88–1.26)	1.52 (0.87–2.66)	1.73 (1.40–2.15)
No	-	-	-	-	-	-
Disability						
With disability	1.27 (0.84–1.94)	1.59 (1.19–2.11)	1.89 (1.36–2.62)	1.45 (1.15–1.82)	1.41 (0.87–2.30)	1.86 (1.42–2.44)
Without disability	-	-	-	-	-	-
Diagnosed physical health condition in past 12 months						
1 or more conditions diag.	1.16 (0.91–1.47)	1.60 (1.37–1.85)	1.35 (1.13–1.62)	1.06 (0.95–1.19)	1.55 (1.18–2.05)	2.09 (1.80–2.43)
None diagnosed	-	-	-	-	-	-
Diagnosed mental health condition in past 12 months						
1 or more conditions diag.	2.70 (2.11–3.47)	2.56 (2.14–3.05)	1.19 (0.95–1.48)	0.91 (0.78–1.06)	1.81 (1.32–2.48)	1.82 (1.52–2.18)
None diagnosed	-	-	-	-	-	-

Logistic regression models, each model is adjusted for relationship status, sexuality and country of birth; models are stratified by age

Among health factors, the odds of each SD increased with poorer self-rated health. Daily pain medication was associated with increased odds of each SD among the 35 to 55 year olds with the exception of reaching climax too quickly and feeling anxious during sex. No association was observed in the 18 to 34 year olds. Disability was associated with increased odds of each SD with the exception of not reaching climax and having trouble getting an erection in the 18 to 34 year olds. A diagnosed physical health condition was associated with increased odds of each SD with the exception of not reaching climax or taking too long in the 18 to 34 year olds and reaching climax too quickly in the 35 to 55 year olds. Being diagnosed with at least one mental health condition in the past 12 months was associated with increased odds of each SD in both age groups, except for reaching climax too quickly, where no association was observed (Tables 2 and 3).

## Discussion

This paper shows that experiencing SD is common among Australian men of the general population – over half the sample (54 %; 95 % CI: 0.53–0.55) experienced at least one SD for more than 3 months in the last 12 months. While more prevalent in older men, almost half the 18 to 24 year old men (48 %) also reported at least one SD highlighting that SD affects men of all ages. Reaching climax too quickly was the most commonly reported SD across all ages (ranging from 32 to 38 %). Depending on age, between 15 and 20 % of men experienced lacking interest, 8 to 12 % experienced lacking enjoyment, 10 to 12 % experienced feeling anxious during sex, 13 to 17 % experienced orgasmic dysfunction, and 10 to 20 % experienced erectile difficulties (ED). Rates for specific SD generally increased with age and were broadly consistent with findings of other Australian and international studies [8, 12, 41, 42].

We found that SDs were associated with both lifestyle and health factors, although the strongest associations were observed for health factors in both age groups, in particular poor self-rated health, having a disability and at least one mental health condition. Poor overall health is consistently found to be associated with SD in men, across all age groups [3, 8, 16, 43, 44]. However, given the cross-sectional nature of our analysis, we do not know the temporal relationship between SD and physical health in our study participants and whether the SD was a consequence of physical health or existed prior to the health conditions. Yet, what is clear is that SDs are common among men with poor physical health, a mental health condition or disability.

SD can also be an indicator of an underlying physical condition. For example, erectile dysfunction has been shown to be a precursor for cardiovascular disease with studies finding that cardiovascular disease is diagnosed within 5 years of development of erectile dysfunction [45]. This highlights the importance of discussing sexual function with a doctor as part of routine or acute health care visits, as it could lead to the diagnosis of an underlying health condition that requires clinical management. However, sexual function is seldom discussed between doctors and patients [46] and research has shown that while embarrassed to initiate sexual health discussions with their doctor, older adults want to be asked and to have the opportunity to discuss their concerns [47, 48].

It has been well established that having a mental health condition is associated with sexual difficulties in men [13, 49–51]. However, this association is complex because anti-depressant and antipsychotic medical treatment can negatively impact on sexual desire, arousal, orgasm and ejaculation problems [35, 52–57], making it difficult to assess whether it is the mental health condition or the treatment or both that is associated with SD. This highlights the importance of discussing SD with any patient being managed for a mental health condition [58]. Practitioners are also advised to revisit and manage these side-effects with their patients on a periodical basis to avoid patients rejecting the treatment and causing worse health outcomes.

The relationship between SD and disability is complicated because it depends on the underlying condition of impairment and its comorbidities [58]. While our findings are consistent with previous studies they display a simplified view of the relationship between disability and SD. However, we were unable to measure specific levels of impairment related to health conditions at this stage of data collection and subsequent longitudinal analyses of Ten to Men will be able to further explore this.

Among lifestyle factors, our findings were consistent with others who have shown that smoking, taking drugs and harmful drinking are associated with SD [59–62]. Smoking tobacco has specifically been linked to erectile difficulties as erectile function relies on normal arterial vascular performance which is adversely affected by smoking [60]. Subsequent analyses with future waves of data collection will investigate the association between smoking and SD in more detail, factoring total exposure (duration and frequency) into the analysis. Poly drug use (marijuana and other drugs) was associated with increased odds of each SD in younger men aged 18 to 34 years, but among older men aged 35 to 55 years, there was less evidence of any association between drug use and lacking interest, lacking enjoyment or feeling anxious during sex. For simplicity we aggregated drug use into three groups separating marijuana from other illicit drugs and our analysis did not capture amount or frequency of drug use which could impact the age groups differently. Future analyses will further investigate the effect of drug use on SD in this cohort.

Obesity was not associated with SDs in the 18 to 34 year olds but showed increased odds for SDs in the older males. It was most strongly associated with erectile dysfunction among older men. This suggests men who continue to be obese may be more likely to develop SD as they age, highlighting that the risk of SD should be discussed with overweight young men as a potential consequence of their health as they age.

Most research into SD is focussed on middle aged and older men (40 years or older) [7, 14, 16, 18, 19] or men with chronic health conditions [58]; very few studies have looked at SD in younger men. Yet, we found that almost half the 18 to 24 year old men reported having experienced at least one SD in the past 12 months for a period of at least 3 months, with over 10 % reporting erectile dysfunction. While alcohol consumption, drug use and smoking have previously been shown to be associated with SD in young men [42, 63], it was a concern that reporting a diagnosed mental health condition was the second most strongly associated factor with SD in young men. A recent longitudinal study of young men did find that men with erectile dysfunction were significantly more likely to develop or maintain depression [42]. It is therefore vital that doctors discuss sexual function with young men early to identify those at risk of mental health conditions.

There are some limitations of this analysis which must be considered when interpreting the data. Firstly, all factors and SD measures were based on self-report and may be subject to reporting bias. Further, we did not ask about the frequency or duration of experiencing a problem or enquire about any distress caused by the problem, necessary factors to fulfil the Diagnostic and Statistical Manual of Mental Disorders definition of sexual dysfunction [64]. As a result, the estimates reported in this paper will overestimate the population-burden of SD among Australian men [65]. Secondly, this was an exploratory analysis in which we investigated the association of several different lifestyle and health factors with SD. As many of these factors are likely to be correlated, we investigated each factor in a separate model. We will undertake more complex analyses to investigate the independent association of these factors with SD in future analyses of the data. Thirdly, these baseline data are cross-sectional and the temporal association between risk factors and SD cannot be confirmed. However, with future waves of data collection in Ten to Men, we will be able to estimate the incidence of SD and investigate temporal relationships between risk factors and SD, allowing us to assess causal associations. Fourthly, although the measures of physical health included in our analysis were based on validated scales, they are summary measures and as a result, we were unable to investigate specific health conditions such as cardiovascular disease or diabetes. Fifthly, several physical health conditions such as mental health may be confounded by medication used to manage the condition and we cannot be certain whether it is the physical condition or the medication that is associated with the SD [3]. Finally, we were unable to exclude men from the analysis who reported no sexual activity in the last 12 months and were unable to relate the experience of SD back to a particular sexual partner; it is possible that the SD reported may only relate to specific sexual partners in the last 12 months or that SD may have prevented men engaging in sex in the last 12 months.

The strengths of this analysis are that we were able to investigate a broad range of lifestyle and health factors for their associations with SDs in males and we were able to investigate whether reporting of SD varied across several different age groups. Our sample size also allowed us to investigate associations with considerable statistical power. Our results will form a baseline for future longitudinal analyses in subsequent waves of data collection.

## Conclusion

Sexual difficulties are common among men of all ages in Australia, but increasingly more prevalent as men grow older. They are strongly associated with both physical and lifestyle factors and can indicate or be a precursor of an underlying physical or mental health condition. Given this, it is imperative that sexual health and sexual functioning be discussed with a doctor as part of the standard health checks and across the lifespan.
